# Mental health and the healthy immigrant effect in Chile: a comparative cross-sectional study with international migrants and locals

**DOI:** 10.3389/fpubh.2025.1582628

**Published:** 2025-08-14

**Authors:** Alice Blukacz, Marcela Oyarte, Báltica Cabieses, Paula Madrid, Alexandra Obach

**Affiliations:** ^1^Centro de Salud Global Intercultural, Facultad de Medicina Clínica Alemana y Facultad de Psicología, Universidad del Desarrollo, Santiago, Chile; ^2^Instituto de Salud Pública, Santiago, Chile; ^3^Department of Health Sciences, University of York, York, United Kingdom

**Keywords:** international migrants, mental health, social determinants of health, Chile, Latin America

## Abstract

**Introduction:**

The question of whether international migrants appear to be in better health than the locals, and whether this “healthy immigrant effect” declines over time is a highly relevant one, especially with regards to mental health. Based on a community-based survey conducted in Santiago, Chile, this study compares the mental health outcomes of international migrants versus local populations and examines differences within the international migrant group of respondents.

**Methods:**

Observational cross-sectional study. Data was collected with international migrants and Chilean participants in 2021–2022 through a structured questionnaire. The study examined self-reported stress and mood disorders in relation to demographic, socioeconomic, health, and migration-related factors. Descriptive analyses were conducted for all variables overall and stratified by perceived stress, mood disorders, and migration status. Associations were assessed using Chi-square or Fisher’s exact tests, with Cramer’s V used to evaluate effect size. Multiple imputation (m = 5) addressed missing data using the mice package in R, followed by generalised logistic regression models fitted across imputed datasets and combined using Rubin’s rules; stepwise selection based on AIC was used for variable reduction, and models were run for the full sample and separately for the migrant population.

**Results:**

The sample included 1,656 international migrants and 1,664 locals. Being a migrant was negatively associated with reporting stress and mood disorders in all analyses. Among migrants, the main risk factors for stress were perceiving a high number of migrants in the neighbourhood and having experienced abuse as a migrant and for mood disorders the main risk factor was reporting having experienced abuse as a migrant as well as a longer stay in Chile.

**Discussion:**

We found a healthy immigrant effect for mental health among international migrants in Chile, which declined over time in the case of mood disorders. Chilean participants reported very high levels of mental health issues, consistent with existing studies. However, results for international migrants highlight both risk and protective factors linked to migration processes, which are unique to them, warranting a specific approach to their mental health needs.

## 1 Introduction

The latest estimate sets the population of international migrants worldwide at 281 million, twice the number it was 1990 (1). The effect of international migration on the health of international migrants is a topic of growing interest, especially with regards to mental health. Migration is recognised as a social determinant of mental health, as it involves processes that interact with other social determinants of health and may amplify existing inequities (2). However, there is inconsistent evidence on whether migration has a positive or negative influence on mental health outcomes, considering that some studies find that international migrants show a higher prevalence of a variety of mental health issues than their local counterparts, while others do not (3–5).

The healthy immigrant effect is a phenomenon where immigrants are generally healthier than native-born populations in their host countries, which may be explained by positive self-selection and cultural buffering (6, 7). This effect has been observed in various countries, but it is not universal (8, 9). Furthermore, the evidence shows that immigrants’ health advantage tends to diminish over time as they might adopt some of the host country unhealthy behaviours (6, 10). With regards specifically to mental health, some studies support the existence of a health immigrant effect as well (11–13). For instance, there are studies showing a mental health advantage among migrants compared to locals, particularly among migrants from countries of origin that are less developed than the country of arrival, and among migrants belonging to ethnic minorities (11, 12, 14).

However, international migrants’ mental health “advantage” may not apply universally across all immigrant groups and can vary by factors such as country of origin and ethnic minority status (4, 15). Notably, some research finds no significant differences between immigrants and native-born individuals, especially for those from English-speaking backgrounds (16). Furthermore, several studies suggest that immigrants’ mental health tends to decline over time (3, 12, 17). Additionally, barriers to mental health care access, including cultural and economic factors, contribute to the underutilization of services by immigrants, and thus, possibly, underdiagnoses and underreporting of mental health issues (18, 19). Despite a potential health immigrant effect, the mental health of international migrants is a cause for concern, as they face a higher likelihood of experiencing adverse situations and stressors that could deteriorate their mental health, such as discrimination, trauma, and social isolation (2). While mental health issues can be developed due to biological preconditions, several socially determined factors influence mental health outcomes, including socioeconomic status, family dynamics, social support, and acculturative stress (13, 20). In this context, migrants may particularly be at risk, especially when facing dangerous migratory trajectories, which can lead to experiencing symptoms of post-traumatic stress disorder, anxiety, and depression (21).

Chile is a high-income country according to the World Bank (22), although it is highly unequal, with 50% of the population earning less than USD600 a month and 16.9% of the total population experiencing multidimensional poverty (23, 24). Regarding mental health indicators, a study conducted in 2019 by the Pan American Health Organisation (PAHO) showed that Chile was among the countries with the worst mental health outcomes in the Americas, as mental health disorders accounted for 2,079.29 Disability-Adjusted Life Years and 2,073.44 Years Lived with Disability per 100.000 population (25). Although some progress has been made to address mental health at the healthcare system level, such as including several mental disorders as part of priority health issues for funding, allowing for easier access to treatment, mental health remains largely insufficiently addressed (26).

Regarding international migration, Chile has experienced a rapid increase in its migrant population. While in 2014, 410,988 international migrants lived in the country, 1,306,859 did in 2018, and the current estimate indicates that 1,918,583 international migrants currently live in Chile, representing almost 10% of the total population (27, 28). The composition of immigration flows also changed, with migrants from Venezuela replacing migrants from Peru as the largest community in Chile from 2018 onwards (29). Most international migrants in Chile are currently from these two countries, followed by Colombian, Haitian, and Bolivian nationals. Although international migrants in Chile are a highly diverse population group, they tend to experience worse socioeconomic conditions than their local counterpart as a whole, and 29.6% experience multidimensional poverty, almost twice as much as the Chilean population (30). Additionally, during the COVID-19 pandemic, an increasing number experienced highly precarious migratory trajectories that included entering the country through non authorised crossing paths by foot and facing the near-impossibility of regularising their migratory status as a result (31).

There are some studies comparing the mental health of international migrants with the locals’ in Chile, and the earliest, conducted in 2007–2008 with migrants mostly from Peru showed a lower prevalence of common mental disorders than among Chileans (32). Another more recent study focused on anxiety and depressive symptoms among Colombian migrants and Chilean nationals also found a higher prevalence of mental health issues among the local population (33). A third study conducted with Peruvian migrants and locals in Chile also found a higher prevalence of common mental disorders among the latter than the former (34). However, other studies highlighted that specific factors could affect the mental health of migrant populations in the country, such as acculturative stress, challenging migratory trajectories, including dangerous conditions of transit to Chile, difficulties to regularise their migratory status, and socioeconomic conditions (35–38). Additionally, these factors may have been exacerbated during the COVID-19 pandemic (39).

In this context, based on a unique community-based survey conducted in the capital city of Santiago, this study first compares the mental health outcomes of international migrants versus local populations and then examines differences within the international migrant group of respondents. The research questions guiding this study are the following: Is there a healthy immigrant effect for mental health among international migrants in Chile? What are the risk and protective factors for mental health among international migrants in Chile?

## 2 Materials and methods

### 2.1 Study design

Observational cross-sectional study, part of the larger research project “Living and health trajectories of international migrants to Chile: how do they compare to the locals and what are their related costs to the healthcare system?” (Fondecyt Regular 1201461 ANID Chile). The main objective of the overall research project is to analyse the trajectories of international migrants to Chile, including living standards, access to and use of healthcare, health status, costs, and consequences of including them to the healthcare system, and compare them to the trajectories of the Chileans over time. The data presented in this article was collected as part of sub study 2 of the project, which is a community-based longitudinal survey of two measures (baseline and follow-up after 1 year). We used the baseline data to conduct this sub analysis focused only on mental health.

### 2.2 Setting and data collection

The study was conducted in three neighbourhoods located in the South-East sector of the capital city of Santiago, Chile: La Pintana, La Granja, and San Ramón, between January 2021 and October 2022. These three neighbourhoods are classified as highly socioeconomically vulnerable, with La Pintana being located at the top of the ranking by social priority index among neighbourhoods of the Metropolitan Region of Santiago, with a score of 88.03, compared to 3.84 for the least socioeconomically vulnerable neighbourhood (40). San Ramón ranked fourth with a score of 82.75 and La Granja ranked thirteenth with a score of 73.89. The neighbourhoods are contiguous and have a total of 391,995 inhabitants (4.6% of the Metropolitan Region) (41). With regards to the international migrant population in the area of interest, data from the Department of Migration of Chile indicates that a little over 1 million migrants live in Santiago (56.8% of the total population of migrants in Chile), and the evidence available estimates that less than 10.000 international migrants live in each of the neighbourhood of interest (42–44). However, the area has welcomed an increasing number of international migrants in the past 10 years. These three neighbourhoods were selected for the study due to their socioeconomic characteristics and the selection was facilitated by an existing partnership with the local public hospital, who expressed interest in the study and the unique data that would be collected.

The data collection instrument was designed based on questions from the 2017 version of the National Socioeconomic Characterisation Survey (*Encuesta de Caracterización Socioeconómica Nacional*, CASEN), the 2017 National Health Survey (*Encuesta Nacional de Salud*, ENS) and other scales validated for migrant populations (45–48). The survey consisted of questions on socio-demographic characteristics, health, use and perception of the healthcare system, income, out-of-pocket expenses, and psychosocial aspects for both populations. In addition, questions on the migration process were asked to the international population.

The survey was about 50-min long and administered through a telephone call or on-site interview in Spanish or Haitian Creole with a trained research assistant, in order to ensure accuracy of data and inclusion of participants with a low level of digital and health literacy. The data is safely stored on the RedCap platform to ensure anonymity and confidentiality.

### 2.3 Participants

The selection criteria for participants were the following: (i) be over 18 years old, (ii) reside in La Pintana, La Granja, or San Ramón (iii) speak Spanish or Creole.

The survey was conducted during the COVID-19 pandemic, which involved two major challenges for participant recruitment: mobility restrictions preventing on-site recruitment and surveying and the need to protect the health of the team and the participants. Furthermore, the international migrant population can be considered “hard-to-reach,” as it can be challenging to involve migrants in health research due to exclusion, marginalisation, and fear of deportation (49). The study included any type of migrant, regardless of their migratory status, based on self-report. In the case of Chilean population considered for this study, barriers to participation were also observed, such as mistrust due to experiences of being scammed being fairly common and lack of time to respond.

Considering these barriers, the research team had to implement a series of remote and onsite (once permitted) recruitment strategies: (1) an invitation to participate in the survey was shared through the social media accounts of municipal institutions, (2) onsite recruitment was conducted in vaccination centres, primary healthcare centres, the waiting room of a hospital, and municipal community centres, (3) onsite recruitment was also conducted in community soup kitchens, street markets and around metro stations and supermarkets. Furthermore, in addition to these strategies aimed at both population groups, the following strategies were used to recruit international migrants: (1) recruiters participated in events organised by migrant-led or pro-migrant organisations, (2) the social worker of the local hospital shared the invitation among her migrant patients. Finally, the snowball technique was implemented throughout the study with both populations.

### 2.4 Variables

The dependent variables were the following:

*Stres*s: This variable was constructed from the question “how often have you felt stressed (irritable, full of anxiety or unable to sleep) during the last year due to situations at home or at work?,” with the following response options: 0 Never felt stress, 1 Sometimes felt stressed at home or at work, 2 Several times felt stressed at home or at work and 3 Permanently felt stressed at home or at work. We recoded as a dichotomous variable: “Stressed,” for those who responded feeling stressed several times or permanently, and “Not stressed” for those who reported never or sometimes feeling stressed.

*Mood disorders:* The variable was constructed from the question “Do you have any of the following chronic illnesses (have you ever been diagnosed by a doctor or taken medication for any of these conditions?), specifically mood disorders (depression, anxiety or other)?,” with the response options Yes, No. The question is focused on self-report based on having been diagnosed and/or treated for a mood disorder, including but not limited to the ones mentioned in the question.

The independent variables were the following:

*Demographic characteristics:* sex (man, woman), age (continuous and by age group 13–18 years old, 19–29 years old, 30–59 years old, 60 years old and over), and marital status (single married or co-habiting partner, divorced or separated, widowed).

*Socio-economic characteristics:* educational level (none or below basic, completed basic education (with or without incomplete secondary education), completed secondary education (with or without incomplete higher education), completed higher education), employment (employed, unemployed, housewife, student or infant), monthly net income (0$, 0$–250.000$, 250.000$–500.000$, 500.000$–1.000.000$, 1.000.000$ or more), and cash benefits during the COVID-19 pandemic (yes, no).

*Housing and surroundings:* tenure of the housing (owner, renter, rented room, other), littering or infestation in the surroundings (yes, no), overcrowding (with, without), visual or acoustic pollution (yes, no), safety (safe, unsafe), and proximity to essential services like pharmacies or supermarkets (yes, no), proximity to a healthcare centre (yes, no).

*Access to healthcare:* health insurance (none, public, private, other or military system, does not know) and barriers to access (yes, no).

*Health status and healthy lifestyles:* alcohol intake (does not drink, drinks less than an average of 2 units a day, drinks more than an average of 2 units a day), physical activity (yes, no), self-reported obesity (yes, no), morbidity (none, treated, untreated), family history of mood disorders (yes, no).

*Psychosocial factors:* perceived importance (“No one cares about what happens to me”- strongly agrees, moderately agrees, moderately disagrees, strongly disagrees) and trust (“It is safer to trust no one”—strongly agrees, moderately agrees, moderately disagrees, strongly disagrees), perceived discrimination (yes, no), having a close friend or group.

*Migration (only for international migrants):* country of origin (Peru, Colombia, Venezuela, Haiti, other), time spent in Chile (number of years), refugee or asylum seeker status (yes, no), perceived number of international migrants in their neighbourhood (0–100), importance of seeking out and maintaining relationships with people from the country of origin (0–100), importance of seeking out and maintaining relationships with people from the country of arrival (0–100), the culture of the country of origin has to be emphasised and contact with the local culture limited (yes, no), feeling that the locals are tolerant towards migrants (yes, no), experiences of abuse as a migrant (yes, no).

### 2.5 Study size

A total of 1,656 international migrants and 1,664 locals were included in this analysis.

Considering that the target population was hard-to-reach, as it was restrained to Chileans and international migrants living in three specific boroughs of the Metropolitan Region of Santiago that concentrate some of the highest levels of multidimensional poverty in the country and a very small proportion of the migrant population in the Metropolitan Region of Santiago, sampling was non probabilistic and non-representative. Given that this is the first study of its kind in the country, the sample size was estimated for categorical variables with maximum uncertainty, an expected proportion of 50%, a confidence level of 95% and an accuracy of 3%, which yielded a minimum number of 1,067 people (epidat 4.1). In accordance with feasibility, the baseline sample size of 1,650 people for each population group was considered adequate and feasible.

### 2.6 Statistical methods

For the total number of respondents, the distribution of each of the variables, alone and according to perceived stress, mood disorders and migration, was described. Additionally, the association between the dependent and independent variables alone was analysed using the Chi-square test (or Fisher’s exact test, as appropriate) in conjunction with Cramer’s V to analyse the magnitude of the association.

To handle missing data in the models, multiple imputation was performed using the mice package in *R. prior* to imputation, the structure of the data was reviewed, and categorical variables were checked to ensure that they were coded as factors, and the nature of missing data was verified. Multiple imputation was carried out with 5 imputed sets (m = 5), using specific methods according to the type of variable: logistic regression for binary variables (logreg), polynomial regression for categorical variables with more than two levels (polyreg), and omitting imputation for the dependent variable perceived stress and mood disorders to avoid introducing bias.

The quality and appropriateness of multiple imputation was assessed by visual diagnosis of convergence of imputation chains through traceplots, comparison of observed and imputed distributions, assessment of the plausibility of imputed values according to the context and logic of the variables, and sensitivity analysis to compare results with and without imputation.

Once the imputation was completed, a generalised logistic regression model (GLM) with logit link was fitted to assess the association between stress (dichotomous) and the independent variables. The models were fitted on each imputed set and the results were combined using Rubin’s rules to obtain estimates and adjusted standard errors. Additionally, selecting an imputation, a GLM model with logistic linkage was fitted and a stepwise step was performed to identify the variables to be included in the model, minimising the AIC (Akaike Information Criterion), which rewards good fit by penalising model complexity, allowing for dimensionality reduction and identification of variables of greater importance for the explanation of the dependent variables of interest (stress and mood disorders in separate models). Once the variables were identified, models were fitted using these sets of variables on each imputed data set and the results were combined using Rubin’s rules. For all models, goodness-of-fit and VIF between the different variables were analysed.

All the above was done both for the total sample and separately for the migrant population only. In the case of the migrant population, GLM models with logit link were additionally fitted, with migration dependent variables.

### 2.7 Ethics statement

The project was approved by the Ethics Committee of the Faculty of Medicine of the Universidad del Desarrollo, as well as the Ethics Committee of the *Servicio de Salud Metropolitano Sur-Oriente.*

The privacy and anonymity of all participants was safeguarded. Participants filled an informed consent form available online through Alchemer before taking part in the survey. Additionally, they could withdraw from the study at any point and refuse to respond any of the questions. Each participant was given a unique ID code linking back to personal data for follow-up purposes and available to the lead researcher and research coordinator only. No individual can be identified from the anonymised dataset.

Each participant was offered approximately USD 5.00 as well as a PDF document was information on access to healthcare, specific services in their borough of residence and support networks upon completion of the questionnaire.

## 3 Results

### 3.1 Sample description

The sample analysed included 3,320 participants, of whom 49.9% (*n* = 1,656) were international migrants and 50.1% (*n* = 1,664) local (born in Chile). The majority of participants were female (72.2%), 30–59 years old (62.6%) and over 55.7% identified themselves as single. The most frequent educational level was completed secondary school (with or without higher education; 39.6%). In terms of employment status, 52% were employed and 24.3% worked as housewives, while 35.1% reported a monthly income of between $250,000 and $500,000 and 29.4% had no declared income. Furthermore, 56.2% reported receiving cash benefits during the SARS-CoV2 pandemic. In relation to housing, 44.5% lived in rented accommodation and 15.6% experienced overcrowding. Although access to nearby healthcare centres (91.6%) and basic services such as pharmacies or supermarkets (98.2%) were most frequently reported, environmental issues were also reported: 62.9% mentioned littering or infestation, and 61.4% mentioned visual or noise pollution in the surrounding area. Despite this, 78.8% considered their surroundings to be safe.

The international migrant participants were mainly Venezuelan (48.2%), most had been living between 1 and 5 years in the country (56.3%), were not refugees (91.5%), had a permanent resident status (27.4%) or had an irregular migratory status (26.2%), and did not travel on their own (54.2%). The majority reported not having given up beliefs and customs (58.0%), considered the locals in Chile to be tolerant (52.2%) and reported not having experienced abuse related to being a migrant (61.2%).

Regarding health, access to healthcare and wellbeing, 49.1% reported experiencing stress, 15.5% reported having a mood disorder and 16.3% reported consuming more than two alcoholic drinks per day. Only 26% were physically active on a regular basis, and 11.6% reported obesity. 20.6% reported having an untreated condition. Additionally, 42.9% faced barriers to accessing the healthcare system, while 12.6% were not affiliated to insurance scheme, showing possible conditions of vulnerability when accessing healthcare services. Regarding psychosocial variables, 35.9% agreed with feeling being important to others, although 30.9% expressed total disagreement with this statement. 57.2% trusted the people around them, and 32.5% reported having experienced discrimination. Finally, 90.9% reported having support networks equivalent to a close group of family and/or friends.

For further detail, please see Supplementary material 1.

### 3.2 Stress and mood disorders among locals and international migrants

Among the respondents self-reporting stress, the stepwise model showed that being female was associated with a higher prevalence of stress (PRR: 1.682; 95%CI: 1.397–2.026), while being married (PRR: 0.704; 95%CI: 0.583–0.848) or cohabiting (PRR: 0.627; 95%CI: 0.477–0.825) was associated with lower prevalence. A lower prevalence of stress was also observed in people with secondary (PRR: 0.695; 95%CI: 0.570–0.847) or higher education (PRR: 0.487; 95%CI: 0.352–0.674) and in those who were physically active (PRR: 0.808; 95%CI: 0.679–0.961) or had a close friend or support group (PRR: 0.659; 95%CI: 0.505–0.859).

In contrast, stress was higher in students or people under 18 (PRR: 1.461; 95%CI: 1.010–2.114), people living with littering or infestation in their surroundings (PRR: 1.373; 95%CI: 1.154–1.632) or with visual or acoustic pollution (PRR: 1.398; 95%CI: 1.177–1.660), those affiliated to the public (PRR: 1.360; 95%CI: 1.058–1.749) or private (PRR: 1.626; 95%CI: 1.032–2.561) healthcare system, who experienced barriers to access healthcare (PRR: 1.825; 95%CI: 1.556–2.139; *p* < 0.001), who consumed more than two alcoholic drinks per day (PRR: 1.260; 95%CI: 1.020–1.558), were under treatment for morbidities (PRR: 1.251; 95%CI: 1.010–1.549), had a family history of mood disorders (PRR: 1.814; 95%CI: 1.511–2.179) or had experienced discrimination (PRR: 1.554; 95%CI: 1.319–1.831; Table 1).

**Table 1:** Stepwise GLM for stress, adjusting for demographic, socioeconomic, healthcare access, health status and psychosocial variables.

Stepwise model for stress			
Term	PRR	CI95%	*p* value
Sex: Woman (ref. Man)	1.682	(1.397 to 2.026)	0.000**
Age 19–29 (13–18)	0.728	(0.352 to 1.506)	0.392
Age 30–59	0.562	(0.270 to 1.170)	0.123
Age 60+	0.540	(0.242 to 1.206)	0.133
Marital status: Married (ref. Single)	0.704	(0.583 to 0.848)	0.000**
Marital status: Cohabiting partner	0.627	(0.477 to 0.825)	0.001**
Marital status: Divorced or separated	1.032	(0.745 to 1.429)	0.851
Marital status: Widowed	0.595	(0.328 to 1.082)	0.089
Educational level: Basic (ref. None)	0.847	(0.684 to 1.048)	0.125
Educational level: Secondary	0.695	(0.570 to 0.847)	0.000**
Educational level: Higher	0.487	(0.352 to 0.674)	0.000**
Employment: Unemployed (ref. Employed)	0.920	(0.744 to 1.138)	0.441
Employment: Housewife	0.881	(0.721 to 1.078)	0.218
Employment: Student or infant	1.461	(1.010 to 2.114)	0.044*
Overcrowding: Yes (ref. No)	0.846	(0.683 to 1.049)	0.127
Littering or infestation in the surroundings: Yes (ref. No)	1.373	(1.154 to 1.632)	0.000**
Visual or acoustic pollution: Yes (ref. No)	1.398	(1.177 to 1.660)	0.000**
Nearby healthcare centre: Yes (ref. No)	0.759	(0.564 to 1.021)	0.068
Healthcare system: Public (ref. None)	1.360	(1.058 to 1.749)	0.016*
Healthcare system: Private	1.626	(1.032 to 2.561)	0.036*
Healthcare system: Other or military	1.019	(0.299 to 3.471)	0.976
Healthcare system: Does not know	1.117	(0.773 to 1.613)	0.555
Barriers to access healthcare: Yes (ref. No)	1.825	(1.556 to 2.139)	0.000**
Barriers to access healthcare: Has never tried	1.085	(0.772 to 1.525)	0.637
Alcohol: Drinks less than an average of 2 units a day (ref. Does not drink)	0.937	(0.769 to 1.141)	0.515
Alcohol: Drinks more than an average of 2 units a day	1.260	(1.020 to 1.558)	0.032*
Physical activity: Yes (ref. No)	0.808	(0.679 to 0.961)	0.016*
Morbidity: Yes, treated (ref. None)	1.251	(1.010 to 1.549)	0.040*
Morbidity: Yes, untreated	1.032	(0.845 to 1.260)	0.760
Family history of mood disorder: Yes (ref. No)	1.814	(1.511 to 2.179)	0.000**
Discrimination: Yes (ref. No)	1.554	(1.319 to 1.831)	0.000**
Having a close friend or support group: Yes (ref. No)	0.659	(0.505 to 0.859)	0.002**
Intercept	0.922	(0.386 to 2.201)	0.855

Model for stress: VIF < 1.62 for all variables; AIC: 4118.4; AUC: 0.7224; Proportion of deviance explained: 0.1154; G^2^ statistic (deviance): 528.616; McFadden’s pseudo-R^2^: 0.1154 R^2^ML (maximum likelihood): 0.1478; R^2^CU (Cragg-Uhler or Nagelkerke): 0.1971. *p value < 0.05; **p value < 0.01.

For mood disorders, the stepwise model showed that being female was associated with a higher prevalence of mood disorders (PRR: 1.996; 95%CI: 1.474–2.701). In contrast, a higher net income was associated with lower prevalence, with the $250,000 to $500,000 category being significant (PRR: 0.625; 95%CI: 0.469–0.833). Living in Type 3 housing (e.g., block flats or similar) was also associated with lower prevalence (PRR: 0.513; 95%CI: 0.307–0.856), as was living in overcrowded conditions (PRR: 0.674; 95%CI: 0.458–0.990), which may reflect contextual or social protective factors. Conversely, having faced barriers to access healthcare (PRR: 1.583; 95%CI: 1.251–2.003), self-reporting obesity (PRR: 2.535; 95%CI: 1.927–3.334), having treated (PRR: 3.421; 95%CI: 2.562–4.570) or untreated morbidities (PRR: 5.726; 95%CI: 4.375–7.493; *p* < 0.001), having family history of mood disorders (PRR: 3.880; 95%CI: 3.084–4.882) and having experienced discrimination (PRR: 1.588; 95%CI: 1.250–2.018), were associated with a higher prevalence of mood disorders (Table 2).

**Table 2:** Stepwise GLM for mood disorders, adjusting for demographic, socioeconomic, healthcare access, health status and psychosocial variables.

Stepwise model for mood disorders			
Term	PRR	CI95%	*p*-value
Sex: Woman (ref. Man)	1.996	(1.474 to 2.701)	0.000**
Net income [$0–$250000] (ref. $0)	0.788	(0.564 to 1.103)	0.164
Net income [$250.000–$500000]	0.625	(0.469 to 0.833)	0.001**
Net income [$500,000–$1000000]	0.712	(0.471 to 1.076)	0.107
Net income above $1,000,000	0.818	(0.334 to 2.003)	0.660
Housing type 2 (ref. Housing type 1)	0.802	(0.617 to 1.042)	0.099
Housing type 3	0.513	(0.307 to 0.856)	0.011*
Housing type 4	0.855	(0.608 to 1.202)	0.366
Overcrowding: Yes (ref. No)	0.674	(0.458 to 0.99)	0.044*
Safety: Yes (ref. No)	1.280	(0.925 to 1.77)	0.136
Barriers to access healthcare: Yes (ref. No)	1.583	(1.251 to 2.003)	0.000**
Barriers to access healthcare: Has never tried	0.525	(0.242 to 1.137)	0.102
Self-reported obesity: Yes (ref. No)	2.535	(1.927 to 3.334)	0.000**
Morbidity: Yes, treated (ref. None)	3.421	(2.562 to 4.57)	0.000**
Morbidity: Yes, untreated	5.726	(4.375 to 7.493)	0.000**
Family history of mood disorder: Yes (ref. No)	3.880	(3.084 to 4.882)	0.000**
Discrimination: Yes (ref. No)	1.588	(1.25 to 2.018)	0.000**
Having a close friend or support group: Yes (ref. No)	0.729	(0.502 to 1.06)	0.098
Intercept	0.027	(0.014 to 0.051)	0.000**

Model for mood disorders: VIF < 1.20 for all variables; AIC: 2095.1; AUC: 0.8545; Proportion of deviance explained: 0.2802; G^2^ statistic (deviance): 800.848; McFadden’s pseudo-R^2^: 0.280; R^2^ML (maximum likelihood): 0.2161; R^2^CU (Cragg-Uhler or Nagelkerke): 0.3722. *p value < 0.05; **p value < 0.01.

Particularly with regards to migration, the results show significant differences between migrants and nationals in terms of perceived stress and mood disorders. 61.1% of nationals reported stress, compared to 37.4% of migrants (*p* < 0.001; Cramér’s V = 0.237), indicating a moderate association. Similarly, nationals reported a higher proportion of mood disorders: 24.4% versus 7% in migrants (χ^2^ = 187.8; *p* < 0.001). This is consistent with the PRRs obtained, and these differences were maintained even after adjusting for demographic, socioeconomic, housing, and environmental variables, access to health care, well-being and psychosocial factors (See Supplementary material 2).

### 3.3 Stress and mood disorders among international migrants

In the specific case of the migrant population, the stepwise model for perceived stress identified multiple significantly associated factors. Being female was associated with a higher prevalence of stress (PRR: 1.437; 95%CI: 1.092–1.890). Being married (PRR: 0.761; 95%CI: 0.586–0.987; *p* = 0.040) or cohabiting (PRR: 0.681; 95%CI: 0.499–0.930) was associated with lower stress compared to being single. Regarding living conditions, living with littering or infestation in the surroundings was associated with higher stress (PRR: 1.296; 95%CI: 1.009–1.664), while having a healthcare centre nearby was associated with lower stress (PRR: 0.630; 95%CI: 0.410–0.969). Being affiliated to the public healthcare system was associated with greater stress (PRR: 1.477; 95%CI: 1.084–2.011), as was experiencing barriers to access healthcare (PRR: 2.022; 95%CI: 1.587–2.575). Being physically active was associated with less stress (PRR: 0.640; 95%CI: 0.492–0.832). Significant associations were also observed with family history of mood disorders (PRR: 2.332; 95%CI: 1.689–3.222) and discrimination (PRR: 1.891; 95%CI: 1.502–2.381).

Regarding the model for mood disorders, those aged 19–29 (PRR: 0.083; 95%CI: 0.015–0.446), 30–59 (PRR: 0.142; 95%CI: 0.028–0.734) and 60 years and older (PRR: 0.071; 95%CI: 0.010–0.528; *p* = 0.010) had a lower prevalence compared to adolescents aged 13–18 years. A higher income was associated with a lower prevalence of mood disorders: for example, those earning between $250,000 and $500,000 (PRR: 0.479; 95%CI: 0.268–0.855) compared with those earning between $500,000 and $1,000,000 (PRR: 0.203; 95%CI: 0.061–0.668). Reporting barriers to access healthcare (PRR: 2.148; 95%CI: 1,337-3,450), obesity (PRR: 2.586; 95%CI: 1.288–5.193), treated (PRR: 3.648; 95%CI: 1.909–6.971) or untreated (PRR: 8.891; 95%CI: 5.211–15.172) morbidities, having a family history of mood disorders (PRR: 6.545; 95%CI: 3.914–10.947), and having experienced discrimination (PRR: 1.642; 95%CI: 1.914–10.947) were factors associated with a higher prevalence of mood disorders (Table 3).

**Table 3:** Stepwise GLM for stress and mood disorders among international migrants, adjusting for demographic, socioeconomic, healthcare access, health status and psychosocial variables.

Term	PRR	CI95%	*p*-value
Stepwise model for stress			
Sex: Woman (ref. Man)	1.437	(1.092 to 1.890)	0.010*
Marital status: Married (ref. Single)	0.761	(0.586 to 0.987)	0.040*
Marital status: Cohabiting partner	0.681	(0.499 to 0.930)	0.016*
Marital status: Divorced or separated	1.122	(0.588 to 2.142)	0.727
Marital status: Widowed	0.278	(0.059 to 1.315)	0.106
Educational level: Basic (ref. None)	2.009	(0.904 to 4.463)	0.087
Educational level: Secondary	1.715	(0.791 to 3.718)	0.171
Educational level: Higher	1.276	(0.567 to 2.875)	0.556
Net income [$0–$250000] (ref. $0)	0.817	(0.560 to 1.191)	0.290
Net income [$250.000–$500000]	0.977	(0.749 to 1.275)	0.864
Net income [$500,000–$1000000]	1.016	(0.626 to 1.649)	0.949
Net income above $1,000,000	0.173	(0.014 to 2.142)	0.164
Littering or infestation in the surroundings: Yes (ref. No)	1.296	(1.009 to 1.664)	0.042*
Visual or acoustic pollution: Yes (ref. No)	1.262	(0.986 to 1.616)	0.065
Nearby healthcare centre: Yes (ref. No)	0.630	(0.410 to 0.969)	0.036*
Healthcare system: Public (ref. None)	1.477	(1.084 to 2.011)	0.013*
Healthcare system: Private	1.973	(0.758 to 5.139)	0.164
Healthcare system: Other or military	0.986	(0.047 to 20.648)	0.993
Healthcare system: Does not know	0.910	(0.546 to 1.516)	0.717
Barriers to access healthcare: Yes (ref. No)	2.022	(1.587 to 2.575)	0.000**
Barriers to access healthcare: Has never tried	1.281	(0.876 to 1.873)	0.201
Alcohol: Drinks less than an average of 2 units a day (ref. Does not drink)	0.805	(0.589 to 1.102)	0.175
Alcohol: Drinks more than an average of 2 units a day	1.359	(0.985 to 1.875)	0.062
Physical activity: Yes (ref. No)	0.640	(0.492 to 0.832)	0.001**
Family history of mood disorder: Yes (ref. No)	2.332	(1.689 to 3.222)	0.000**
Discrimination: Yes (ref. No)	1.891	(1.502 to 2.381)	0.000**
Having a close friend or support group: Yes (ref. No)	0.739	(0.528 to 1.035)	0.078
Intercept	0.238	(0.088 to 0.638)	0.004**
Stepwise model for mood disorders			
Age 19–29 (ref. 13–18)	0.083	(0.015 to 0.446)	0.004**
Age 30–59	0.142	(0.028 to 0.734)	0.020*
Age 60+	0.071	(0.010 to 0.528)	0.010*
Net income [$0–$250000] (ref. $0)	0.934	(0.485 to 1.801)	0.838
Net income [$250.000–$500000]	0.479	(0.268 to 0.855)	0.013*
Net income [$500,000–$1000000]	0.203	(0.061 to 0.668)	0.009**
Net income above $1,000,000	0.579	(0.056 to 5.960)	0.646
Overcrowding: Yes (ref. No)	0.576	(0.323 to 1.029)	0.062
Visual or acoustic pollution: Yes (ref. No)	1.548	(0.978 to 2.450)	0.062
Supermarket or pharmacy nearby: Yes (ref. No)	0.323	(0.093 to 1.124)	0.076
Barriers to access healthcare: Yes (ref. No)	2.148	(1.337 to 3.450)	0.002**
Barriers to access healthcare: Has never tried	0.748	(0.301 to 1.858)	0.532
Self-reported obesity: Yes (ref. No)	2.586	(1.288 to 5.193)	0.008**
Morbidity: Yes, treated (ref. None)	3.648	(1.909 to 6.971)	0.000**
Morbidity: Yes, untreated	8.891	(5.211 to 15.172)	0.000**
Family history of mood disorder: Yes (ref. No)	6.545	(3.914 to 10.947)	0.000**
Discrimination: Yes (ref. No)	1.642	(1.035 to 2.604)	0.035*
Intercept	0.337	(0.049 to 2.325)	0.269

Model for stress: VIF < 1.37 for all variables; AIC: 2030.8; AUC: 0.7026; Proportion of deviance explained: 0.0943; G^2^ statistic (deviance): 205.610; McFadden’s pseudo-R^2^: 0.0943 R^2^ML (maximum likelihood): 0.1172; R^2^CU (Cragg-Uhler or Nagelkerke): 0.1598. Model for mood disorders: VIF < 1.21 for all variables; AIC: 610.61; AUC: 0.8827; Proportion of deviance explained: 0.3106; G^2^ statistic (deviance): 258.938; McFadden’s pseudo-R^2^: 0.311; R^2^ML (maximum likelihood): 0.1458; R^2^CU (Cragg-Uhler or Nagelkerke): 0.3664. *p value < 0.05; **p value < 0.01.

Considering only variables related to migration, the stepwise model for perceived stress in the migrant population identified several significantly associated variables. A higher perceived number of migrants in the neighbourhood was associated with a slight but significant increase in stress (PRR = 1.006; 95%CI: 1.002–1.009). Regarding the attitude towards the statement “The culture of the country of origin has to be emphasised and contact with the local culture limited” (reference: ‘Strongly disagree’), those who said they “disagreed” were less likely to report stress (PRR = 0.666; 95%CI: 0.456–0.971), as were those who “agreed” (PRR = 0.581; 95%CI: 0.398–0.848). Feeling that the local population is tolerant of migrants was also associated with a lower likelihood of stress (PRR = 0.768; 95%CI: 0.616–0.958). In contrast, having experienced abuse due to being a migrant was associated with a significantly increased risk of stress, doubling the odds (PRR = 2.087; 95%CI: 1.684–2.586).

For mood disorders, two statistically significant associations were observed. First, length of residence in Chile was positively associated with mood disorders (PRR = 1.048; 95%CI: 1.003–1.095; *p* = 0.036), suggesting a progressive increase in risk as length of stay increases. In addition, having experienced abuse due being a migrant was associated with an increased risk, more than twice as high compared to those who had not (PRR = 2.161; 95%CI: 1.463–3.193; *p* < 0.001). Although differences by country of origin were not statistically significant, Haitian migrants tended to have a lower risk (PRR = 0.417; 95%CI: 0.173–1.003; *p* = 0.051). In both cases there was no significant collinearity between the variables (Table 4).

**Table 4:** Stepwise GLM for stress and mood disorders among international migrants, adjusting for migration’s variables.

Term	PRR	CI95%	*p*-value
Stepwise model for stress			
Perceived amount of international migrants in their neighbourhood	1.006	(1.002 to 1.009)	0.001**
The culture of the country of origin has to be emphasised and contact with the local culture limited (ref. Strongly disagree)			
Disagree	0.666	(0.456 to 0.971)	0.035*
Neutral	0.801	(0.611 to 1.049)	0.107
Agree	0.581	(0.398 to 0.848)	0.005**
Strongly agree	0.788	(0.497 to 1.250)	0.312
Feels that the locals are tolerant towards migrants (ref. No)			
Yes	0.768	(0.616 to 0.958)	0.019*
Has experienced abuse for being a migrant (ref. No)			
Yes	2.087	(1.684 to 2.586)	0.000**
Intercept	0.440	(0.331 to 0.586)	0.000**
Stepwise model for mood disorders			
Country of origin (ref. Peru)			
Colombia	1.095447	(0.520 to 2.308)	0.810
Venezuela	1.0821981	(0.567 to 2.067)	0.811
Haiti	0.4168935	(0.173 to 1.003)	0.051
Other	1.344578	(0.688 to 2.629)	0.387
Time spent in Chile (years)	1.0478481	(1.003 to 1.095)	0.036*
Has experienced abuse for being a migrant (ref. No)			
Yes	2.1614391	(1.463 to 3.193)	0.000**
Intercept	0.0432037	(0.022 to 0.084)	0.000**

Model for stress: VIF < 1.05 for all variables; AIC: 2117.2; AUC: 0.627; Proportion of deviance explained: 0.03633; G^2^ statistic (deviance): 79.22; McFadden’s pseudo-R^2^: 0.036; R^2^ML (maximum likelihood): 0.047; R^2^CU (Cragg-Uhler or Nagelkerke): 0.064. Model for mood disorders: VIF < 1.59 for all variables; AIC: 816.28; AUC: 0.6524; Proportion of deviance explained: 0.0375; G^2^ statistic (deviance): 31.26; McFadden’s pseudo-R^2^: 0.0375; R^2^ML (maximum likelihood): 0.0188; R^2^CU (Cragg-Uhler or Nagelkerke): 0.047. *p-value < 0.05; **p-value < 0.01.

## 4 Discussion

This study aimed at comparing mental health outcomes between international migrants and locals, as well as examining differences among international migrants, in three neighbourhoods of the Metropolitan Region of Santiago, Chile. The analysis was conducted with data collected through a community-based survey during 2021–2022, as part of a larger project focusing on migration and health in Chile. Considering the data available and the social determinants of health approach, mental health was conceptualised as reported stress and diagnosis of mood disorders and independent variables related to demographic and socioeconomic characteristics, living conditions, access to healthcare, health status and healthy lifestyles, psychosocial factors, and, for migrants, factors related to the migratory process.

Regarding the comparison between international migrants and locals, we consistently found that being a migrant was negatively associated with stress and mood disorders, which is aligned with previous quantitative studies comparing the mental health of both populations in Chile (32–34) and suggesting a healthy immigrant effect. However, it is important to take three elements into account. The first one is that the Chilean population reported high rates of stress and mood disorders. With regards to stress, the only recent study conducted in Chile, based on a non-representative survey with a sample of 500 people, indicated that 73% of the respondents reported being stressed to the point of it affecting their daily lives and 54% to the point of having to stop working for a while (50). Regarding mood disorders, the latest National Health survey conducted in 2016–2017 found that 15.8% of the population had depressive symptomatology and that the prevalence of depression was 6% (51). This, together with the data from PAHO presented in the introduction (25), indicates that mental health issues are a top health concern for the Chilean population, potentially overshadowing the mental health issues experienced by the international migrant population in the country.

The second point is that our results are based on self-report rather than diagnosis, which could introduce both information and recall biases due to a variety of factors such as health literacy or diverse interpretations of mental health issues in a context of cultural diversity, leading participants to either underestimate or overestimate their levels of stress or mental health conditions, affecting the accuracy of the estimates. However, self-reports have been found to be correlated with posteriorly diagnosed mental health issues (52). Further studies including variables such as health literacy, as well as validated diagnosis tools may be relevant to further explore the healthy immigrant effect among international migrants in Chile.

The third point is that, our analysis found that length of residence in Chile was positively associated with reporting mood disorders, suggesting that the healthy immigrant effect declines over time, which is consistent with the existing literature on the topic (3, 12, 17). This may be due, on the one hand, to improved access to healthcare and diagnosis as barriers related to migratory status, information, and cross-cultural communication may fade (53), or, on the other hand, as an actual decline in mental health over time while being exposed to similar risk factors as the Chilean population. Further longitudinal studies taking into account these factors may be relevant in order to determine the risk factors over time.

While the international migrants surveyed in this study generally presented better health outcomes than their Chilean counterpart, it is still relevant to examine their risk factors for stress and mood disorders, especially the ones related to migration. Concerning stress, perceiving a high number of migrants in the neighbourhood was a statistically significant risk factor which may be counter-intuitive, however, it may be a proxy for territorial exclusion and residential segregation, where migrant populations are concentrated, over time, in more socioeconomically deprived areas (54, 55). This may be especially the case as this study was conducted in three neighbourhoods that are classified as highly vulnerable and that have seen an increased number of international migrants in recent years.

Another noteworthy risk factor for both stress and mood disorders was having experienced abuse as a migrant, which is consistent with other studies conducted in Chile, the United States, Norway, and Spain (56–59). Furthermore, perceiving that the locals are tolerant towards international migrants was a protective factor for stress, indicating the importance of considering the wider social dynamics at play in the everyday life of international migrants and their impact on mental health. This is especially important in a climate of growing hostility towards migrant populations worldwide, and in particular in Chile. While migration was not a politicised topic until the mid 2010s, anti-immigrant sentiment has been growing at the national level, fuelled by the media and discriminatory political discourses in the past few years (60, 61).

Our results are relevant for healthcare practitioners, as factors related to migration are key to understanding potential mental health challenges among international migrant patients, from an intercultural point of view. Understanding the risk factors specific to international migrants allows for mental healthcare that is specific and responsive to their needs, contributing to improving their mental health outcomes. Furthermore, understanding protective factors is also key for mental health promotion, as is recognising the resources and strategies international migrants have to support their mental health. More widely, considering that discrimination and having experienced abuse as a migrant were risk factors for stress and mood disorders, while perceiving that the locals are tolerant was a protective factor for stress, wider action must be taken regarding the xenophobia experienced by international migrants in Chile. Policies aiming at improving the perception and attitudes of local populations towards immigrants must be implemented, especially targeting school-age children and areas with a high density of migrant population.

This study has some limitations. The data was collected during the pandemic in highly challenging conditions with “hard-to-reach” populations, limiting the possibility of a representative sample. Additionally, this limited our ability to reach populations facing a higher degree of social vulnerability and barriers to healthcare and essential services. The non-probabilistic sampling, the presence of non-response, and the restriction to three municipalities in the country may limit the external validity of the findings, meaning that the results cannot be generalised to the entire migrant or local population in Chile. Additionally, the questions included in the survey focused mainly on experiences in the country of arrival, leaving aside questions regarding pre-migration stress or other specific questions linked to family (or other issues) in the home country. The cross-sectional nature of the study prevents the establishment of causal relationships, and some of the selected variables may have bidirectional associations with stress and mood disorders. Furthermore, regarding the design of the study, although performing a mixed-methods study in order to complement the quantitative data presented in this article may have been relevant and is increasingly recognised as useful in the field of epidemiology (62), this study contributes to the existing evidence with a unique set of community-based data collected in three highly vulnerable neighbourhoods of Santiago, Chile with both international migrants and Chileans. There are existing qualitative studies on the mental health of international migrants and refugees in Chile (36, 37, 39) and further studies may explore the topic through mixed methods. In all, this data gives us insights into understudied and underserved populations, potentially contributing to strategies aiming at improving their mental health outcomes.

This study adds to the existing evidence on the healthy immigrant effect and confirms that there is a healthy immigrant effect for mental health in Chile. However, considering that international migrants in Chile still face mental issues, albeit in a lesser proportion than their local counterparts, and that results highlight both risk and protective factors linked to migration, we may ask the question of what matters most for practice and policymaking: that international migrants have better mental health outcomes than locals, or that their mental health is influenced by mechanisms related to migratory processes?

## Data Availability

The raw data supporting the conclusions of this article will be made available by the authors, without undue reservation.

## References

[1] McAuliffe FM OuchoBrien LA. World migration report 2024. Geneva: International Organization for Migration (IOM) (2024).

[2] World Health Organization. Mental health of refugees and migrants: Risk and protective factors and access to care. Geneva: WHO (2023). 37972226

[3] Elshahat S Moffat T Newbold KB. Understanding the healthy immigrant effect in the context of mental health challenges: a systematic critical review. J Immigr Minor Health. (2021) 24:1564–79. doi: 10.1007/s10903-021-01313-5, PMID: 34807354 PMC8606270

[4] Islam F. Examining the" healthy immigrant effect" for mental health in Canada. Univ Tor Med J. (2013) 90:169–75.

[5] Close C Kouvonen A Bosqui T Patel K O’Reilly D Donnelly M. The mental health and wellbeing of first generation migrants: a systematic-narrative review of reviews. Glob Health. (2016) 12:1–13. doi: 10.1186/s12992-016-0187-3, PMID: 27558472 PMC4997738

[6] Kennedy S Kidd MP McDonald JT Biddle N. The healthy immigrant effect: patterns and evidence from four countries. J Int Migr Integr. (2015) 16:317–32. doi: 10.1007/s12134-014-0340-x

[7] Domnich A Panatto D Gasparini R Amicizia D. The “healthy immigrant” effect: does it exist in Europe today? Ital J Public Health. (2012) 9:e7532-3. doi: 10.2427/7532

[8] Vang ZM Sigouin J Flenon A Gagnon A. Are immigrants healthier than native-born Canadians? A systematic review of the healthy immigrant effect in Canada. Ethn Health. (2017) 22:209–41. doi: 10.1080/13557858.2016.1246518, PMID: 27809589

[9] Constant AF García-Muñoz T Neuman S Neuman T. A “healthy immigrant effect” or a “sick immigrant effect”? Selection and policies matter. Eur J Health Econ. (2018) 19:103–21. doi: 10.1007/s10198-017-0870-1 28144758

[10] Nolan A Layte R. The ‘healthy immigrant effect’: breastfeeding behaviour in Ireland. Eur J Pub Health. (2014) 25:626–31. doi: 10.1093/eurpub/cku177, PMID: 25422364

[11] Dhadda A Greene G. ‘The healthy migrant effect’ for mental health in England: propensity-score matched analysis using the EMPIRIC survey. J Immigr Minor Health. (2018) 20:799–808. doi: 10.1007/s10903-017-0570-z, PMID: 28389831 PMC6061089

[12] Rivera B Casal B Currais L. Length of stay and mental health of the immigrant population in Spain: evidence of the healthy immigrant effect. Appl Econ. (2015) 47:1972–82. doi: 10.1080/00036846.2014.1002895

[13] Alegría M Álvarez K DiMarzio K. Immigration and mental health. Curr Epidemiol Rep. (2017) 4:145–55. doi: 10.1007/s40471-017-0111-2, PMID: 29805955 PMC5966037

[14] Montazer S. Economic development of origin-countries, life-stage at immigration, and length of residence effects on psychological distress. Social Currents. (2018) 5:583–604. doi: 10.1177/2329496518780922

[15] Lee R. Does the healthy immigrant effect apply to mental health? Examining the effects of immigrant generation and racial and ethnic background among Australian adults. SSM-Population Health. (2019) 7:100311. doi: 10.1016/j.ssmph.2018.10.011, PMID: 31294073 PMC6595271

[16] Straiton M Grant JF Winefield HR Taylor A. Mental health in immigrant men and women in Australia: the north West Adelaide health study. BMC Public Health. (2014) 14:1111. doi: 10.1186/1471-2458-14-1111, PMID: 25349060 PMC4228163

[17] Mason J Laporte A McDonald JT Kurdyak P Fosse E de Oliveira C. Assessing the “healthy immigrant effect” in mental health: intra-and inter-cohort trends in mood and/or anxiety disorders. Soc Sci Med. (2024) 340:116367. doi: 10.1016/j.socscimed.2023.116367, PMID: 38039769

[18] Blukacz A Cabieses B Markkula N. Inequities in mental health and mental healthcare between international immigrants and locals in Chile: a narrative review. Int J Equity Health. (2020) 19:1–15. doi: 10.1186/s12939-020-01312-2, PMID: 33148258 PMC7640394

[19] Moore L Jayaweera H Redshaw M Quigley M. Migration, ethnicity and mental health: evidence from mothers participating in the millennium cohort study. Public Health. (2019) 171:66–75. doi: 10.1016/j.puhe.2019.03.022, PMID: 31103615

[20] Sirin SR Sin E Clingain C Rogers-Sirin L. Acculturative stress and mental health: implications for immigrant-origin youth. Pediatr Clin N Am. (2019) 66:641–53. doi: 10.1016/j.pcl.2019.02.010, PMID: 31036240

[21] Virupaksha HG Kumar A Nirmala BP. Migration and mental health: an interface. J Nat Sci Biol Med. (2014) 5:233–9. doi: 10.4103/0976-9668.136141, PMID: 25097389 PMC4121889

[22] World Bank. GNI per capita, PPP (current international $). Chile: World Bank (2022).

[23] Instituto Nacional de Estadísticas. Síntesis de resultados Encuesta Suplementaria de Ingresos. Santiago: INE (2023).

[24] Ministerio de Desarrollo Social y Familia. CASEN 2022 Pobreza Multidimensional. Santiago: Ministerio de Desarrollo Social y Familia (2023).

[25] Pan American Health Organization. ENLACE DATA Portal The Burden of Mental Disorders. US: PAHO (2025).

[26] Vergara G Dembowski N Cruz C. Garantías y Garantías Explícitas en Salud (GES-AUGE) en patología de Depresión en el sistema de salud chileno. 10 años de experiencia y aprendizaje. PSIQUIATRÍA Y SALUD MENTAL. (2017) 34:5–20.

[27] Departamento de Extranjería y Migración. Migración en Chile 2005–2014. Santiago: DEM (2015).

[28] Instituto Nacional de Estadísticas. Estimación de personas extranjeras 2023. Santiago, Chile: Instituto Nacional de Estadísticas (2023).

[29] Instituto Nacional de Estadísticas, Departamento de Extranjería y Migración. Estimación de Personas Extranjeras Residentes en Chile al 31 de Diciembre 2018. Santiago: INE y DEM (2019).

[30] Servicio Nacional de Migraciones. Pobreza y migración Desde la Casen 2022. Santiago: SERMIG (2023).

[31] Stefoni C Cabieses B Blukacz Á. Migraciones y COVID-19: Cuando el discurso securitista amenaza el derecho a la salud. Simbiótica Revista Eletrônica. (2021) 8:38–66. doi: 10.47456/simbitica.v8i2.36378

[32] Rojas G Fritsch R Castro A Guajardo V Torres P Díaz B. Trastornos mentales comunes y uso de servicios de salud en población inmigrante. Rev Med Chile. (2011) 139:1298–304. doi: 10.4067/S0034-98872011001000008, PMID: 22286729

[33] Urzúa A Caqueo-Urízar A Aragón D. Prevalencia de sintomatología ansiosa y depresiva en migrantes colombianos en Chile. Rev Med Chile. (2020) 148:1271–8. doi: 10.4067/S0034-98872020000901271, PMID: 33399702

[34] Errazuriz A Schmidt K Valenzuela P Pino R Jones PB. Common mental disorders in Peruvian immigrant in Chile: a comparison with the host population. BMC Public Health. (2023) 23:1274. doi: 10.1186/s12889-023-15793-7, PMID: 37391769 PMC10314508

[35] Urzúa A Heredia O Caqueo-Urízar A. Salud mental y estrés por aculturación en inmigrantes sudamericanos en el norte de Chile. Rev Med Chile. (2016) 144:563–70. doi: 10.4067/S0034-98872016000500002 27552005

[36] Carreño A Blukacz A Cabieses B Jazanovich D. “Nadie está preparado para escuchar lo que vi”: atención de salud mental de refugiados y solicitantes de asilo en Chile. Salud Colect. (2021) 16:e 3035. doi: 10.18294/sc.2020.3035 33374087

[37] Blukacz A Carreño Calderon A Obach A Cabieses B Peroncini J Oliva A. Perceptions of health needs among Venezuelan women crossing the border in northern Chile during the COVID-19 pandemic. Int J Environ Res Public Health. (2022) 19:15175. doi: 10.3390/ijerph192215175, PMID: 36429892 PMC9690325

[38] Carreño-Calderón A Cabieses B Correa-Matus ME. Individual and structural barriers to Latin American refugees and asylum seekers' access to primary and mental healthcare in Chile: a qualitative study. PLoS One. (2020) 15:e0241153. doi: 10.1371/journal.pone.0241153, PMID: 33156878 PMC7647080

[39] Blukacz A Cabieses B Obach A Madrid P Carreño A Pickett KE. “If I get sick here, I will never see my children again”: the mental health of international migrants during the COVID-19 pandemic in Chile. PLoS One. (2022) 17:e0277517. doi: 10.1371/journal.pone.0277517, PMID: 36445885 PMC9707751

[40] Seremi de Desarrollo Social y Familia RM. Índice de prioridad social de comunas 2022. Santiago, Chile: Ministerio de Desarrollo Social y Familia (2022).

[41] Biblioteca del Congreso Nacional de Chile. Reported Comunales. Santiago: BCN (2024).

[42] Servicio Nacional de Migraciones. Minuta población migrante en la comuna de San Ramón. Santiago: SERMIG (2024).

[43] Servicio Nacional de Migraciones. Minuta población migrante en la comuna de La Pintana. Santiago: SERMIG (2024).

[44] Servicio Nacional de Migraciones. Minuta población migrante en la comuna de La Granja. Santiago: SERMIG (2024).

[45] Mitchell PH Powell L Blumenthal J Norten J Ironson G Pitula CR. A short social support measure for patients recovering from myocardial infarction: the ENRICHD social support inventory. J Cardiopulm Rehabil Prev. (2003) 23:398–403. doi: 10.1097/00008483-200311000-00001, PMID: 14646785

[46] Arenas P Urzúa A. Estrategias de aculturación e identidad étnica: un estudio en migrantes sursur en el norte de Chile. Univ Psychol. (2016) 15:117–28. doi: 10.11144/Javeriana.upsy15-1.eaie

[47] Eshel Y Rosenthal-Sokolov M. Acculturation attitudes and sociocultural adjustment of sojourner youth in Israel. J Soc Psychol. (2000) 140:677–91. doi: 10.1080/00224540009600509, PMID: 11195720

[48] Marin G Sabogal F Marin BV Otero-Sabogal R Perez-Stable EJ. Development of a short acculturation scale for Hispanics. Hisp J Behav Sci. (1987) 9:183–205.

[49] Van Den Muijsenbergh M Teunissen E van Weel-Baumgarten E Van Weel C. Giving voice to the voiceless: how to involve vulnerable migrants in healthcare research. Br J Gen Pract. (2016) 66:284–5. doi: 10.3399/bjgp16X685321, PMID: 27231286 PMC4871282

[50] IPSOS Global Advisor. Día Mundial de la Salud Mental 2024 Informe LATAM IPSOS (2024).

[51] Ministerio de Salud. ENCUESTA NACIONAL DE SALUD 2016–2017 Segunda entrega de resultados. Santiago: MINSAL (2018).

[52] Santos BF Oliveira HN Miranda AES Hermsdorff HHM Bressan J Vieira JCM. Research quality assessment: reliability and validation of the self-reported diagnosis of depression for participants of the cohort of universities of Minas Gerais (CUME project). J Affective Disorders Reports. (2021) 6:100238. doi: 10.1016/j.jadr.2021.100238

[53] Lin S. Healthy immigrant effect or under-detection? Examining undiagnosed and unrecognized late-life depression for racialized immigrants and nonimmigrants in Canada. J Gerontol: Series B. (2024) 79:gbad104. doi: 10.1093/geronb/gbad104, PMID: 37498769 PMC11036341

[54] Benassi F Iglesias-Pascual R Salvati L. Residential segregation and social diversification: exploring spatial settlement patterns of foreign population in southern European cities. Habitat Int. (2020) 101:102200. doi: 10.1016/j.habitatint.2020.102200

[55] Galeano J Bayona-i-Carrasco J. Residential segregation and clustering dynamics of migrants in the metropolitan area of Barcelona: a demo-spatial analysis at the census tract level. Quetelet J. (2018) 6:99–127. doi: 10.14428/rqj2018.06.01.05

[56] Straiton ML Aambø AK Johansen R. Perceived discrimination, health and mental health among immigrants in Norway: the role of moderating factors. BMC Public Health. (2019) 19:325. doi: 10.1186/s12889-019-6649-9, PMID: 30894173 PMC6425660

[57] Szaflarski M Bauldry S. The effects of perceived discrimination on immigrant and refugee physical and mental health. Immigration Health. (2019) 19:173–204. doi: 10.1108/S1057-629020190000019009 PMC6553658 31178652

[58] Llácer A Del Amo J Garcia-Fulgueiras A Ibanez-Rojo V Garcia-Pino R Jarrin I. Discrimination and mental health in Ecuadorian immigrants in Spain. J Epidemiol Community Health. (2009) 63:766–72. doi: 10.1136/jech.2008.085530, PMID: 19416929

[59] Ubach LI Santacana FI. Discriminación percibida, afrontamiento y salud mental en migrantes peruanos en Santiago de Chile. Psicoperspectivas. (2016) 15:157–68. doi: 10.5027/psicoperspectivas-Vol15-Issue1-fulltext-613

[60] Stefoni C Brito S. Migraciones y migrantes en los medios de prensa en Chile: La delicada relación entre las políticas de control y los procesos de racialización. Rev Hist Soc Ment. (2019) 23:1–28. doi: 10.35588/rhsm.v23i2.4099

[61] Sibrian Díaz ND Alfaro A Núñez JC. Validación de instrumento sobre exposición de comunidades migrantes a discursos de odio en el ecosistema mediático chileno: resultados preliminares. Rev Lat Comun Soc. (2024) 82:42. doi: 10.4185/rlcs-2024-2226

[62] Houghton LC Paniagua-Avila A. Why and how epidemiologists should use mixed methods. Epidemiology. (2023) 34:175–85. doi: 10.1097/EDE.0000000000001565, PMID: 36722799 PMC9891266

